# Diagnostic Accuracy of Point-of-Care Gram Stains in Obstructive Pyelonephritis due to Ureteral Stones

**DOI:** 10.1093/ofid/ofae026

**Published:** 2024-02-02

**Authors:** Satoshi Hayano, Toshiya Hidaka, Risako Tadakuma, Masayuki Kashima

**Affiliations:** Department of Internal Medicine, Japanese Red Cross Kumamoto Hospital, Kumamoto, Japan; Department of Clinical Laboratory, Japanese Red Cross Kumamoto Hospital, Kumamoto, Japan; Department of Clinical Laboratory, Japanese Red Cross Kumamoto Hospital, Kumamoto, Japan; Department of Internal Medicine, Japanese Red Cross Kumamoto Hospital, Kumamoto, Japan

**Keywords:** Gram stain, obstructive pyelonephritis, point-of-care testing, ureteral stones, urinary tract infection

## Abstract

**Background:**

The diagnostic utility of point-of-care (POC) Gram stains for obstructive pyelonephritis with hydronephrosis is not well established. The current study aimed to assess the diagnostic accuracy of urine Gram stains in patients with obstructive pyelonephritis due to ureteral stones.

**Methods:**

A retrospective observational study was conducted on patients with obstructive pyelonephritis admitted to our hospital between January 2011 and December 2021. The diagnostic accuracy of Gram stains was evaluated based on the severity of hydronephrosis, including Gram stains performed by both trained physicians and microbiological technicians.

**Results:**

After analyzing 210 patients, POC Gram stains of bladder urine presented a sensitivity, specificity, positive predictive value, and negative predictive value of 86.8%, 81.8%, 93.7%, and 66.7%, respectively, for gram-negative rods and 65.7%, 83.4%, 48.9%, and 91.0%, respectively, for gram-positive cocci. The agreement between POC Gram stains and urine culture was good for gram-negative rods, with a kappa (κ) coefficient of 0.637 and agreement rate of 85.6%, and moderate for gram-positive cocci, with a κ coefficient of 0.435 and agreement rate of 80%. The agreement between POC Gram stains and bladder urine culture results for gram-negative rods was higher in the mild hydronephrosis group (κ coefficient = 0.677) than in the severe hydronephrosis group (κ coefficient = 0.466). Discrepancies in Gram stain results between physicians and technicians were observed in 21 of 180 cases (11.7%).

**Conclusions:**

POC Gram stains for gram-negative rods may be a useful diagnostic tool for obstructive pyelonephritis, particularly in cases of mild hydronephrosis.

Obstructive pyelonephritis is a rapidly progressive condition in which 20.8%–41% of cases develop septic shock [1–5]. Mortality rates for obstructive pyelonephritis range from 2% to 12%, escalating significantly in patients with septic shock [2, 4, 6]. Given that diagnostic errors, delayed antimicrobial administration, and delayed ureteral drainage have all been associated with increased mortality, early diagnosis is crucial [7–9].

As a form of point-of-care (POC) testing, urine Gram stains have been proven invaluable for the prompt diagnosis of urinary tract infection (UTI) [10, 11]. POC Gram stain (PCGS) of urine showed a moderate correlation and high agreement with urine culture in both uncomplicated and complicated UTI [10]. Despite limited data on clinical efficacy, previous studies have indicated that PCGS for both uncomplicated and complicated UTIs yields comparable treatment success rates to guideline-based therapy while also reducing the cost of broad-spectrum antimicrobials [12].

Nonetheless, severe ureteral obstruction can cause false-negative outcomes in bladder urine (BU) cultures [13, 14]. In a previous study, BU cultures had a sensitivity and specificity of 30.2% and 73% for a positive renal pelvic urine (RPU) culture, respectively, and BU and RPU cultures had low concordance in asymptomatic bacteriuria with ureteral obstruction [14]. In addition, blood and RPU cultures were concordant in 66.7% of patients who developed bacteremia after urologic procedures, whereas all BU cultures were negative [14]. These findings suggest potential problems with the sensitivity of BU cultures for patients with ureteral obstruction. However, the mechanism by which ureteral obstruction affects Gram stains in obstructive pyelonephritis remains unclear.

The current study aimed to elucidate the diagnostic characteristics of Gram stains for BU and RPU in patients with obstructive pyelonephritis attributable to ureteral calculi and to investigate the impact of hydronephrosis.

## METHODS

### Study Design and Participants

This single-center, retrospective, observational study evaluated the diagnostic accuracy of POC and laboratory Gram stains in patients with obstructive pyelonephritis. The study was conducted between January 2011 and December 2021 at the Japanese Red Cross Kumamoto Hospital, a 490-bed teaching hospital in Kumamoto City, Japan. Several patients in the emergency department (ED) who were diagnosed by ED or general internal medicine physicians and urologists were visited. A majority of patients were admitted to the general internal medicine department for treatment.

The eligibility criteria were as follows: (1) patients diagnosed with obstructive pyelonephritis with ureteral stones; (2) patients aged ≥18 years; and (3) patients with Gram stains from either BU or RPU. To promote data independence, only the first episode in patients with multiple episodes of obstructive pyelonephritis during the study period was included. The exclusion criteria were as follows: (1) patients who died within 48 hours of admission; (2) those who had received inpatient treatment at another hospital; (3) those who received prior antibiotic treatment for >24 hours; and (4) those without hydronephrosis. We excluded patients who died within 48 hours of presenting to the hospital due to inability to gather appropriate urine Gram stain and culture data, since most of these patients were so ill upon arrival that they were quickly admitted to palliative care without further diagnostic workup.

### Data Collection and Case Definitions

Data were extracted from the electronic medical record of each patient. The following baseline participant demographics were collected: age, sex, location of infection (nosocomial, hospital-acquired, and healthcare-associated), intensive care unit admission, acute kidney injury, Charlson Comorbidity Index score, drainage method (double-J stent, extracorporeal shock wave lithotripsy [ESWL], nephrostomy), history of prior antibiotic therapy, presence of sepsis and septic shock, obstruction site, stone location and size (maximum diameter), degree of hydronephrosis, and complications related to infection [1, 2–4]. Sepsis and septic shock were defined according to the criteria provided in the Third International Consensus Definitions for Sepsis and Septic Shock (Sepsis-3) [15].

Pyelonephritis was diagnosed based on clinical symptoms (eg, fever, chills, urinary frequency, dysuria, and back pain) and urinalysis and culture findings (eg, pyuria and bacteriuria) [5]. In patients with only fever as a symptom, pyelonephritis was diagnosed after excluding other sources of infection, confirming pyuria or bacteriuria, or identifying a predominant organism in the urine culture. Obstructive pyelonephritis due to ureteral stones was defined as urinary tract obstruction caused by ureteral stones diagnosed via computed tomography or abdominal ultrasound and associated with hydronephrosis. Hydronephrosis was classified based on the Society for Fetal Urology (SFU) grading system, which has 4 grades (I–IV) [16, 17]. Accordingly, SFU grades I–II indicate mild hydronephrosis, whereas SFU grades III–IV indicate severe hydronephrosis. With regard to appropriate empirical and definitive therapy, treatment was defined as appropriate if antibiotics to which the causative organisms were susceptible were started for 24 hours or longer.

### Urine Gram Stains and Urinalysis

Bladder urine samples were collected using the midstream clean-catch urine collection procedure or disposable catheters with an aseptic technique. In addition, pelvic urine was collected aseptically from the catheter during double-J stent or nephrostomy insertion. Uncentrifuged urine samples were Gram stained using the Bartholomew and Mittwer (B&M) modified method with neo-B&M Wako (Fujifilm Wako Pure Chemical Corporation, Japan), a 4-step staining procedure using crystal violet, 2% iodine sodium hydroxide, acetone ethyl alcohol, and 0.1% fuchsin; or the Favor method with Faber G “Nissui” (Shimadzu Diagnostics Corporation, Japan), a 3-step staining procedure using 0.2% Victoria Blue, 2% picric acid ethanol, and 0.004% fuchsin. Slides were examined using light microscopy at both 100-fold and 1000-fold magnification under oil immersion.

BU and RPU Gram stains were performed and interpreted by either physicians or microbiological technicians and were considered positive when an average of approximately 1 bacterial cell per field was observed at 1000-fold magnification. Gram stains were defined as positive if any organisms were observed and as negative if no organisms were detected. Morphotypes and the presumptive bacteria were as follows: gram-negative rods for Enterobacterales, *Pseudomonas aeruginosa*, *Acinetobacter baumannii*, and *Bacteroides fragilis*; gram-positive cocci for *Enterococcus faecalis*, *Enterococcus faecium*, *Streptococcus* spp, *Staphylococcus* spp, and *Aerococcus urinae*; and gram-positive rods for *Corynebacterium* spp and *Actinomyces* spp.

Physician-assisted PCGS was performed immediately after specimen collection by junior residents (postgraduate year [PGY] 1–2) and general internal medicine physicians (PGY 3–5) who were supervised by attending physicians. Junior residents receive education on Gram staining as part of the core curriculum in medical education, and general internal medicine physicians undergo additional Gram stain training during their residency.

Laboratory Gram stains were checked by microbiological technicians with a minimum of 3–15 years of experience, and PCGS were checked by general internal medicine physicians with a minimum of 1–3 years of on-the-job clinical training. Urine cultures were performed by laboratory technicians on aseptically collected urine. Bacterial quantification was performed using BD BBL CHROMagar Orientation agar medium (Becton, Dickinson and Company, Franklin Lakes, New Jersey). We included in the analysis any clinically and microbiologically significant pathogens that were identified, regardless of whether the bacterial count from the quantitative urine culture was <10^4 ^colony-forming units (CFU)/mL [8].

Identification and susceptibility testing were performed using VITEK-2 (bioMérieux Japan Ltd, Tokyo, Japan). Urine culture contamination was determined when normal vaginal flora grew in the urine culture. In addition, coagulase-negative staphylococci (eg, *Staphylococcus epidermidis*, *Staphylococcus capitis*), *Staphylococcus aureus*, *Corynebacterium* spp, and α-streptococci were considered causative organisms only if identified in at least 1 set of blood cultures with the same organism as the urine culture or 2 sets of blood cultures. Urinalysis was performed by laboratory technicians using the Uriflet S (ARKRAY Factory, Inc, Shiga, Japan) for qualitative testing. Pyuria, hematuria, and nitrate were defined as positive when the qualitative urinalysis yielded a result of 1+ or higher.

### Blood Cultures

Before antibiotic administration, at least 2 sets of blood cultures were performed using the BD BACTEC FX Blood Culture System, BD BACTEC Aerobic/23F Culture Vials, and BD BACTEC Lytic Anaerobic/23F Culture Vials (Nippon Becton Dickinson Company, Ltd, Tokyo, Japan). The broth microdilution method was used to determine the minimum inhibitory concentrations of the antibiotics. The susceptibility of the isolated organisms to different antibiotics was evaluated according to the Clinical and Laboratory Standards Institute (CLSI 2010) M100-S20.

### Data Analysis

Categorical variables were analyzed using the χ^2^ or Fisher exact test, whereas continuous variables were analyzed using the Student *t* test. For all statistical tests, 2-tailed *P* values <.05 were considered statistically significant. BU and RPU Gram stains were compared to BU and RPU urine cultures as the reference standard for calculating sensitivity, specificity, positive predictive value (PPV), negative predictive value (NPV), and positive and negative likelihood ratios (LRs) with 95% confidence intervals. Kappa (κ) statistics were also used to evaluate the agreement between the results of BU and RPU Gram stains and those of urine cultures. Statistical analyses were performed using Stata version 15.1 software (StataCorp, College Station, Texas).

### Patient Consent Statement

This study was approved by the ethics committee of the Japanese Red Cross Kumamoto Hospital (approval number 523), which waived the need to obtain informed consent.

## RESULTS

### Baseline Characteristics

During the study period, 297 patients were enrolled and a total of 87 patients were excluded based on the following criteria: no hydronephrosis (n = 25), transfer from other hospitals (n = 18), no Gram stain performed by physicians at POC (n = 17), prior antibiotics ≥2 days (n = 10), infection occurred after stone drainage (n = 9), death within 48 hours (n = 3), age <18 years (n = 3), or no admission (n = 2). Many of the patients who die very early after emergency presentation for calculous pyelonephritis have irreversible organ damage at the time of presentation and are given emergency advanced care planning and a palliative treatment plan with minimal testing and treatment. Many of the patients who died within 48 hours at our institution died early after presentation to the ED (in fact, the 3 patients who died within 48 hours all died within 24 hours of arrival at the hospital, and were given a palliative treatment policy and were not adequately examined). We decided to exclude these patients from the study because it was often impossible to collect the necessary data for the study.

Baseline characteristics are shown in Table 1. Mild and severe hydronephrosis were observed in 173 and 37 patients, respectively. The median stone diameter was 8.6 mm (interquartile range [IQR], 6.3–12.1 mm), with a minimum and maximum of 1.5 and 84 mm, respectively. The most common drainage method was double-J catheter placement, and the median time from admission to insertion of double-J catheters was 4 hours (IQR, 2.7–6 hours), placement of a nephrostomy tube was 10.3 hours (IQR, 9.5–21.6 hours), and performing ESWL was 113 hours (IQR, 73–144 hours).

**Table 1.** Baseline Characteristics

Characteristic	Total	Mild Hydronephrosis	Severe Hydronephrosis
	(N = 210)	(n = 173)	(n = 37)
Age, y, median (IQR)	74.0 (59.0–84.0)	74.0 (60.0–84.0)	70.0 (53.0–82.0)
Female sex	154 (73.3)	127 (73.4)	27 (73.0)
Location of infection			
Community acquired	156 (74.3)	128 (74.0)	28 (75.7)
Nosocomial	7 (3.3)	5 (2.9)	2 (5.4)
Healthcare associated	47 (22.4)	39 (22.5)	8 (21.6)
ICU care	59 (28.1)	49 (28.3)	10 (27.0)
Acute kidney injury	118 (56.2)	102 (59.0)	16 (43.2)
Sepsis	106 (50.5)	90 (52.0)	16 (43.2)
Septic shock	40 (19.0)	33 (19.1)	7 (18.9)
Charlson Comorbidity Index score, median (IQR)	0.0 (0.0–1.0)	0.0 (0.0–1.0)	1.0 (0.0–2.0)
Stone size, mm, median (IQR)	8.6 (6.3–12.1)	8.0 (6.0–12.0)	11.0 (8.8–13.0)
Hydronephrosis (SFU grade)			
1	101 (48.1)	101 (58.4)	0 (0.0)
2	72 (34.3)	72 (41.6)	0 (0.0)
3	32 (15.2)	0 (0.0)	32 (86.5)
4	5 (2.4)	0 (0.0)	5 (13.5)
Obstruction side			
Right	94 (44.8)	75 (43.4)	19 (51.4)
Left	113 (53.8)	95 (54.9)	18 (48.6)
Bilateral	3 (1.4)	2 (1.2)	1 (2.7)
Stone location			
Renal pelvis	18 (8.6)	15 (8.7)	3 (8.1)
Ureteropelvic junction	23 (11.0)	19 (11.0)	4 (10.8)
Proximal ureter	103 (49.0)	85 (49.1)	18 (48.6)
Middle ureter	19 (9.0)	15 (8.7)	4 (10.8)
Distal ureter	23 (11.0)	16 (9.2)	7 (18.9)
Vesicoureteral junction	27 (12.9)	25 (14.5)	2 (5.4)
Drainage (n = 116)			
Double-J stent	103 (88.8)	81 (90.0)	22 (84.6)
ESWL	8 (6.9)	6 (6.7)	2 (7.7)
Nephrostomy	5 (4.3)	3 (3.3)	2 (7.7)
Urinalysis (n = 206)			
Pyuria	176 (85.4)	145 (83.8)	31 (83.8)
Nitrate	66 (32)	54 (31.2)	12 (32.4)
Hematuria	188 (91.3)	157 (90.8)	31 (83.8)
Proteinuria	178 (86.4)	146 (84.4)	32 (86.5)
pH, median (IQR)	6.0 (5.5–7.0)	6.0 (5.5–7.0)	6.0 (5.5–7.0)
Urine and blood culture			
Positive urine culture (BU)	188 (89.5)	158 (91.3)	30 (81.1)
Polymicrobial urine culture (BU)	34 (16.2)	24 (13.9)	10 (27.0)
Positive urine culture (RPU)	82 (39.0)	63 (36.4)	19 (51.4)
Polymicrobial urine culture (RPU)	9 (9.2)	7 (9.3)	2 (8.7)
Positive blood culture	127 (60.5)	111 (64.2)	16 (43.2)
Polymicrobial blood culture	4 (3.1)	4 (3.6)	0 (0.0)
Appropriate empirical therapy	182 (86.7)	149 (86.1)	33 (89.2)
Appropriate definitive therapy	199 (94.8)	166 (96.0)	33 (89.2)
Antimicrobial treatment, d, median (IQR)	19.5 (15.0–22.0)	21.0 (15.0–22.0)	16.0 (14.0–21.0)
Prior antibiotic use	36 (17.2)	30 (17.4)	6 (16.2)

Data are presented as No. (%) unless otherwise indicated.

Abbreviations: BU, bladder urine; ESWL, extracorporeal shock wave lithotripsy; ICU, intensive care unit; IQR, interquartile range; RPU, renal pelvic urine; SFU, Society of Fetal Urology.

There were 4 deaths (2.2%) within 28 days, with 1 patient with progressive multiorgan failure due to septic shock and another patient within 1 week of admission due to ventricular fibrillation as a complication of heart failure after improvement of infection. The remaining 2 patients recovered from the infection but died due to worsening renal failure. Infection-related complications included 2 cases of renal abscess and 1 case of osteomyelitis due to bacteremia from a UTI, both observed in the mild hydronephrosis group.

### BU and RPU Gram Stains

Point-of-care Gram stains for BU and RPU were performed in 180 of 210 patients (85.7%) and 50 of 98 patients (51.0%), respectively, whereas laboratory Gram stains were performed in all cases.

Table 2 shows the sensitivity, specificity, PPV, NPV, positive LR, and negative LR of Gram stains for BU and RPU. The details of the 2 × 2 contingency table are provided in Supplement 1. The characteristics of the BU Gram stains, stratified according to hydronephrosis severity, are detailed in Supplement 2.

**Table 2.** Diagnostic Property and Concordance Rates of Gram Stain for Bladder Urine and Renal Pelvic Urine

Gram Stain	Sensitivity, %	Specificity, %	PPV, %	NPV, %	Positive LR	Negative LR	κ Coefficient	Agreement, %
GNR-positive group in urine culture								
BU								
Gram stain in POC	86.8 (79.9–92)	81.8 (67.3–91.8)	93.7 (87.9–97.2)	66.7 (52.5–78.9)	4.77 (2.54–8.96)	0.16 (.1–.25)	0.637	85.6
Gram stain in laboratory	89.9 (84.2–94.1)	94.1 (83.0–98.8)	97.9 (94.1–99.6)	75 (62.6–85)	15.3 (5.09–45.9)	0.1 (.07–.17)	0.774	91
RPU								
Gram stain in POC	84.6 (69.5–94.1)	81.8 (48.2–97.7)	94.3 (80.8–99.3)	60 (32.3–83.7)	4.65 (1.32–16.4)	0.19 (.09–.41)	0.588	84
Gram stain in laboratory	87.8 (78.2–94.3)	91.7 (73–99)	97 (89.6–99.6)	71 (52–85.8)	10.5 (2.79–39.8)	0.13 (.07–.25)	0.724	88.8
GPC positive group in urine culture								
BU								
Gram stain in POC	65.7 (47.8–76.4)	83.4 (76.4–89.1)	48.9 (34.1–63.9)	91 (84.8–95.3)	3.97 (2.57–6.15)	0.41 (.26–.65)	0.435	80
Gram stain in laboratory	88.6 (75.4–96.2)	90.4 (84.8–94.4)	70.9 (57.1–82.4)	96.8 (92.6–98.9)	9.2 (5.7–14.8)	0.13 (.06–.29)	0.724	90
RPU								
Gram stain in POC	62.5 (24.5–91.5)	83.3 (68.6–93)	41.7 (15.2–72.3)	92.1 (78.6–98.3)	3.75 (1.58–8.89)	0.45 (.18–1.11)	0.381	80
Gram stain in laboratory	76.9 (46.2–95)	94.1 (86.8–98.1)	66.7 (38.4–88.2)	96.4 (89.8–99.2)	13.1 (5.31–32.2)	0.25 (.09–.66)	0.667	91.8

Data in parentheses indicate the 95% confidence interval.

Abbreviations: BU, bladder urine; GNR, gram-negative rod; GPC, gram-negative cocci; LR, likelihood ratio; NPV, negative predictive value; POC, point–of–care; PPV, positive predictive value; RPU, renal pelvic urine.

PCGS for BU was negative in 37 of the 180 patients (20.6%). Among these patients, additional RPU Gram stains came back positive in 8 of the 37 patients (21.6%). BU cultures were positive in 20 of the 37 patients (55%) with negative PCGS for BU, with 11 of these 20 patients (55%) having a bacterial count <10^5 ^CFU/mL (urine culture bacterial count: 10^1 ^CFU/mL, 1 patient; 10^2 ^CFU/mL, 1 patient; 10^3 ^CFU/mL, 10 patients; 10^5 ^CFU/mL, 10 patients; 10^6 ^CFU/mL, 2 patients). PCGS for BU was negative in 10 of the 31 patients (32.3%) with severe hydronephrosis and 27 of the 149 patients (18.1%) with mild hydronephrosis (*P* = .08). PCGS for BU was negative in 8 of the 31 patients (16%) with prior antibiotic administration and 29 of the 149 patients (19.7%) without prior antibiotic administration (*P* = .43).

In addition, PCGS for RPU was negative in 8 of 50 cases (16%). Among these 8 cases, 5 (66.7%) had a positive RPU culture (1 case with 10^2 ^CFU/mL, 3 cases with 10^3 ^CFU/mL, and 1 case with 10^6 ^CFU/mL). All patients with a negative PCGS for RPU received antibiotics prior to drainage, with the median time from antibiotic administration to drainage being 2 hours (IQR, 0.9–7.0 hours). Only 1 patient received antibiotics prior to visitation.

Discrepancies between POC and laboratory test results were observed in 21 of the 180 cases (11.7%) who underwent BU Gram stains and 5 of the 50 cases (10%) who underwent RPU Gram stains. For BU samples, the distribution of causative bacteria in discrepant cases included 11 cases of gram-negative rods (GNRs), 11 cases of gram-positive cocci (GPC), and 2 cases of gram-positive rods (GPRs). Regarding GPC, 8 of the 11 cases had multiple bacteria in the urine (GNR + GPC), with only GNR being determined as positive Gram stains by physicians. However, the number of bacteria in the BU culture was ≤10^4 ^CFU/mL for GPC. Misidentification of Gram stain properties and morphology occurred in 2 of the 22 cases. Specifically, the physician incorrectly identified 1 case of GPR (*Corynebacterium* spp) as GPC. In another case, the physician misidentified GPC (*E faecalis*) as GNR. RPU samples were additionally submitted in 6 of the 22 discrepant BU Gram stains cases, all of which were correctly identified by the physician.

For RPU Gram stains, the physician found negative results in 3 of the 5 cases, whereas the technician identified GNR as positive, with the results being consistent with those of culture. In 2 cases, only the physician determined GNR to be positive, with the results being consistent with those of culture. There were only 3 cases in which PCGS for both BU and RPU were negative, and the number of bacteria in both BU and RPU cases was ≤10^3 ^CFU/mL.

We also determined the diagnostic accuracy of the Gram stains according to the number of bacteria in the urine culture (Supplement 3), excluding polymicrobial infections. PCGS for BU showed low sensitivity and concordance with BU urine cultures, especially for bacterial counts ≤10^3 ^CFU/mL. However, the sensitivity of the Gram stains increased when bacterial counts were ≥10^4 ^CFU/mL. For GPC, the sensitivity of the Gram stains decreased significantly when the bacterial count in the urine culture was ≤10^3 ^CFU/mL. When classifying patients according to the severity of hydronephrosis, we found that the severe hydronephrosis group had significantly more patients with a low bacterial count (≤10^3 ^CFU/mL) in BU cultures than did the mild hydronephrosis group (42/149 [28.2%] vs 13/26 [50%]; *P* = .03). Furthermore, among the 35 cases with bacterial counts of ≤10^3 ^CFU/mL in BU culture, 22 (62.9%) demonstrated positive blood culture results concordant with the causative pathogens identified in BU culture.

Table 3 shows the results of urinalysis. The sensitivity and specificity of nitrite were analyzed only in the population with GNR. Among the 206 BU samples, pyuria and nitrite were positive in 176 (85.4%) and 66 (32%) cases, respectively. Among the 30 cases who tested negative for pyuria, 15 (50%) and 21 (70%) had positive PCGS for BU and BU culture, respectively.

**Table 3.** Diagnostic Property of Urinalysis

Urinalysis	Sensitivity, %	Specificity, %	PPV, %	NPV, %	Positive LR	Negative LR
Pyuria	88.3 (82.8–92.5)	54.5 (32.2–75.6)	94.3 (89.8–97.2)	35.3 (19.7–53.5)	1.94 (1.23–3.08)	0.22 (.12–.37)
Nitrate	35.5 (28.6–42.9)	95.2 (76.2–99.9)	98.5 (91.8–100)	14.5 (9.08–21.5)	7.46 (1.09–51)	0.68 (.59–.78)

Data in parentheses indicate the 95% confidence interval.

Abbreviations: CI, confidence interval; LR, likelihood ratio; NPV, negative predictive value; PPV, positive predictive value.

### Urine and Blood Cultures

Table 4 shows the results of blood and urine cultures. BU culture was submitted for 210 cases (mild vs severe hydronephrosis: 173 vs 37 cases), and RPU culture was submitted for 98 cases (mild vs severe hydronephrosis: 75 vs 23 cases).

**Table 4.** Bacteria Isolated From Bladder and Renal Pelvic Urine Cultures

Bacterial Species	Isolated From BU	MDR	Isolated From RPU	MDR	Isolated From Blood Culture	MDR
Gram-negative rod	173 (76.9)	…	79 (84.9)	…	104 (80)	…
*Escherichia coli*	112 (49.8)	ESBL: 23 (20.5)	52 (55.9)	ESBL: 10 (19.2)	66 (50.8)	ESBL: 16 (24.2)
*Klebsiella pneumoniae*	14 (6.2)	ESBL: 2 (14.3)	9 (9.7)	ESBL: 1 (11.1)	14 (10.8)	ESBL: 2 (14.3)
*Klebsiella oxytoca*	1 (0.4)	…	1 (1.1)	…	1 (0.8)	…
*Proteus mirabilis*	16 (7.1)	…	6 (6.5)	…	10 (7.7)	…
*Proteus vulgaris*	3 (1.3)	…	2 (2.2)	…	2 (1.5)	…
*Enterobacter cloacae*	2 (0.9)	…	1 (1.1)	…	2 (1.5)	…
*Enterobacter aerogenes*	4 (1.8)	AmpC: 1 (25)	2 (2.2)	…	3 (2.3)	…
*Citrobacter freundii*	1 (0.4)	…	0	…	0	…
*Citrobacter koseri*	2 (1.2)	…	1 (1.1)	…	2 (1.5)	…
*Citrobacter farmeri*	1 (0.4)	…	0	…	0	…
*Serratia marcescens*	3 (1.3)	…	0	…	1 (0.8)	…
*Hafnia alvei*	1 (0.4)	…	1 (1.1)	…	1 (0.8)	…
*Edwardsiella tarda*	1 (0.4)	…	0	…	0	…
*Pseudomonas aeruginosa*	9 (4)	…	4 (4.3)	…	2 (1.5)	…
*Acinetobacter baumannii*	1 (0.4)	…	0	…	0	…
*Bacteroides fragilis*	1 (0.4)	…	0	…	0	…
Unidentified	1 (0.4)	…	0	…	0	…
Gram-positive cocci	46 (20.4)	…	14 (15.1)	…	24 (18.5)	…
*Enterococcus faecalis*	27 (12)	…	8 (8.6)	…	11 (8.5)	…
*Enterococcus faecium*	2 (0.9)	…	1 (1.1)	…	2 (1.5)	…
*Peptostreptococcus*	6 (2.7)	…	3 (3.3)	…	3 (2.3)	…
α-streptococci	1 (0.4)	…	1	…	1 (0.8)	…
*Aerococcus urinae*	0	…	0	…	1 (0.8)	…
*Streptococcus dyagalactiae*	1 (0.4)	…	0	…	0	…
*Streptococcus agalactiae*	6 (2.7)	…	0	…	1 (0.8)	…
*Staphylococcus epidermidis*	0	…	0	…	4 (3.1)	…
*Staphylococcus capitis*	0	…	1 (1.1)	…	1 (0.8)	…
CoNS (unidentified)	3 (1.3)	…	0	…	0	…
Gram-positive rod	6 (2.7)	…	0	…	2 (1.5)	…
*Actinomyces odontolyticus*	0	…	0	…	1 (0.8)	…
*Corynebacterium* spp	4 (1.8)	…	0	…	1 (0.8)	…
Unidentified	2 (0.9)	…	0	…	0	…

Data are presented as No. (%).

Abbreviations: BU, bladder urine; CoNS, coagulase-negative staphylococci; ESBL, extended-spectrum β-lactamase; MDR, multidrug resistant; RPU, renal pelvic urine.

Among the bacteria isolated from BU, GNRs, GPC, and GPRs accounted for 173 (76.9%), 46 (20.4%), and 6 cases (2.7%), respectively. *Escherichia coli* was the most common organism detected in both urine and blood cultures, with 20.5% of the *E coli* in BU being extended-spectrum β-lactamase (ESBL)–producing bacteria. In BU samples, *E faecalis* was the most common GPC detected (27/46 cases [58.7%]). *Enterococcus faecium* was found in only 2 cases. No vancomycin-resistant enterococci were detected.

Polymicrobial infections were observed in 34 of the 210 BU samples (16.2%), 9 of the 98 RPU samples (9.2%), and 3 cases who underwent blood culture (3.1%). Blood culture findings showed 1 case of *E coli* (ESBL) + *Klebsiella pneumoniae*, 1 case of *E coli* (ESBL) + *Streptococcus agalactiae*, and 1 case of *E coli* (ESBL) + *E faecium*, all of which matched the causative bacteria in the BU culture.

In 3 of 210 cases (1.4%), BU showed positive Gram stains but negative BU cultures, with 1 case having received prior antibiotic treatment. In RPU, 5 of 98 cases (5.1%) showed positive Gram stains with negative RPU cultures, with 3 cases having received prior antibiotic treatment before drainage.

### Antimicrobial Therapy

Table 5 shows the results of antimicrobial therapy. The most commonly used antibiotic was cefotiam, the second-generation cephalosporin with a spectrum similar to cefuroxime. It was administered as empirical therapy in 62 cases, of which 88.7% were determined to be an appropriate therapy. Of the 18 cases where the empirical therapy was inappropriate, 14 were changed to broad-spectrum antibiotics based on the results of urine cultures. The results of the urine cultures at the time of changing antibiotics was as follows: 5 cases were *E coli* (ESBL), 5 cases were *Enterobacter* spp, 3 cases were *E coli* (non-ESBL), and 1 case was *P aeruginosa*. For definitive therapy, the most commonly used antibiotic was ampicillin administered in 69 cases, with 100% determined to be appropriate therapy.

**Table 5.** Initial and Definitive Treatment Antibiotics

Antibiotic	Total, No.	Appropriate, No.	Inappropriate, No.	Rate of Appropriate Therapy, %
Initial treatment antibiotics, No.	n = 253	n = 210	n = 43	
Cefotiam	62	55	7	88.7
Cefmetazole	8	8	0	100
Ceftriaxone or cefotaxime	30	22	8	73.3
Ceftazidime	4	1	3	25
Ampicillin	6	5	1	83.3
Ampicillin-sulbactam	39	32	7	82.1
Piperacillin	1	0	1	0
Piperacillin-tazobactam	37	31	6	83.8
Meropenem	24	22	2	91.7
Vancomycin	8	8	0	100
Teicoplanin	1	1	0	100
Gentamicin	8	8	0	100
Tobramycin	25	25	0	100
Definitive treatment antibiotics, No.	n = 225	n = 216	n = 9	
Cefazolin	18	18	0	100
Cefotiam	39	36	3	92.3
Cefmetazole	19	19	0	100
Ceftriaxone or cefotaxime	19	19	0	100
Ceftazidime	4	4	0	100
Ampicillin	69	69	0	100
Ampicillin-sulbactam	15	14	1	93.3
Piperacillin	10	10	0	100
Piperacillin-tazobactam	7	6	1	85.7
Meropenem	5	5	0	100
Aztreonam	1	1	0	100
Levofloxacin	1	1	0	100
Vancomycin	5	1	4	20
Gentamicin	3	3	0	100
Tobramycin	9	9	0	100

Combination therapy was administered in 39 of the 210 cases (18.6%) among the patients receiving empirical therapy. The antimicrobial agents used in combination therapy were aminoglycosides in 32 of the 210 cases (15.2%), vancomycin in 6 of the 210 cases (2.9%), and teicoplanin in 1 of the 210 cases (0.5%). In patients receiving definitive therapy, combination therapy was administered in 11 of the 210 cases (5.2%), with aminoglycosides used in 8 of the 210 cases (3.8%) and vancomycin in 3 of the 210 cases (1.4%).

## DISCUSSION

This retrospective observational study assessed the characteristics of Gram stains in cases of obstructive pyelonephritis due to ureteral stones. To our knowledge, this is the first published study investigating the accuracy of BU and RPU sample Gram stains specifically in obstructive pyelonephritis.

Our study found strong correlation between BU PCGS and cultures for GNRs. A previous study on complicated pyelonephritis, wherein 10% of patients had calculous pyelonephritis caused by GNRs, found that PCGS had a sensitivity of 95.4%, specificity of 87.2%, and κ coefficient of 0.608 (95% confidence interval, .571–.653) [10]. This suggests that PCGS may also be valuable in the diagnosis of complicated pyelonephritis [10]. However, both Gram stains’ sensitivity for GNRs and concordance rate with urine culture were reduced in the severe hydronephrosis group. This necessitates particular caution when interpreting PCGS for BU results in patients with severe hydronephrosis.

The decreased sensitivity of BU Gram stains among patients with severe hydronephrosis could have been attributed to the low bacterial counts in the urine caused by ureteral obstruction. The severe hydronephrosis group included in this study had significantly more patients with low bacterial counts (≤10^3 ^CFU/mL) in BU culture. In fact, 1 study showed that urine Gram stains have high reliability when the bacterial count is ≥10^5 ^CFU/mL in urine culture but may show decreased sensitivity when the bacterial count is ≤10^3 ^CFU/mL [18]. In our study, good agreement rates were observed in cases with bacterial counts ≥10^4 ^CFU/mL. In general, bacterial counts >10^4^ CFU/mL are considered significant in urine culture. However, in cases with complicated pyelonephritis, infection can be established even with bacterial counts of ≤10^3^ CFU/mL, indicating a lower threshold for infection [18–20]. This study showed that even with bacterial counts of ≤10^3^ CFU/mL in the urine culture, 62.9% of the cases had a positive blood culture. This indicates that bacteremia may occur in cases with obstructive pyelonephritis despite having low bacterial counts in the urine. Therefore, checking multiple fields of view during a Gram stain and conducting double-checks with laboratory technicians are crucial. These findings suggest that physicians should be cautious about quickly changing or discontinuing antibiotics in suspected cases of obstructive pyelonephritis, as negative PCGS or low bacterial counts in urine cultures can be misleading.

In our study, *Enterococcus* spp emerged as the second most common causative organism (13.0%), consistent with findings presented in previous studies suggesting a range of 8.0%–19.7% [6, 21]. As with a previous study, the concordance rate between PCGS and urine culture in BU samples, particularly sensitivity, was lower for GPC than for GNRs [10]. The most frequent cause of the reduced sensitivity was oversight given that physicians tended to overlook GPC more often than GNRs, especially in cases with polymicrobial infections. While the reasons for the overlooking of GPC remain uncertain, we infer that the trend in UTI may be influenced by the predominance of GNRs as the primary pathogens, potentially leading physicians to inadvertently overlook the presence of GPC. However, only 1 of the 33 cases (3.0%) with *Enterococcus* infection tested negative for GPC in both BU and RPU PCGS. Notably, this particular case had a low bacterial count in the urine culture at 10^3^ CFU/mL. If GPC are not identified in both BU and RPU Gram stains, reconsideration of the empirical treatment for *Enterococcus* spp might be warranted.

Most discrepancies between physicians and laboratory technicians involved overlooking a small number of bacteria. PCGS performed by physicians could be expected to have minimal discrepancies with laboratory Gram stains in cases of obstructive pyelonephritis with mild hydronephrosis. Therefore, caution should be exercised in situations involving severe hydronephrosis, polymicrobial infections, and low bacterial counts given the decreased sensitivity of PCGS in these circumstances.

Although only 14.6% of the cases had negative leukocyte esterase (LE) tests, 50% of those with a negative LE test had a positive PCGS for BU. Given that urine LE and urine nitrite tests alone are insufficient for diagnosis, studies have shown that adding urine Gram stains could potentially increase the positivity rates [11]. Moreover, adding Gram stains in cases who tested negative for LE may increase the possibility of diagnosing obstructive pyelonephritis. In LE-negative cases, not all patients with positive Gram stains are clinically significant, so contamination must also be considered. However, in our study, there was only 1 instance where GPRs, suggestive of *Lactobacillus* spp, were observed on Gram stains.

The important roles of a PCGS in the diagnosis and treatment of UTIs extend to prompt diagnosis, prediction of etiological bacteria, cost efficiency, and reduced use of broad-spectrum antibiotics without decreasing treatment success rates [10, 12, 22]. Additionally, sometimes Gram stains can provide information even in cases with negative cultures. Currently, only a few studies have been published on the clinical effectiveness of Gram stain–based treatment of complicated pyelonephritis, and current guidelines offer no explicit recommendations in this regard. Antimicrobial resistance frequently presents a challenge in the treatment of complicated pyelonephritis. As suggested by reports from Japan, the incidence of ESBL-producing *E coli* as causative bacteria in complicated pyelonephritis has increased annually, ranging from 15.2% to 24.3% over the past decade [21]. In broad-spectrum antibiotics, meropenem is effective against ESBL-producing bacteria, which are common in Japan. However, piperacillin-tazobactam is not recommended for the treatment of complicated UTI against ESBL-producing bacteria [23]. In our study, 16.2% of initial therapy with piperacillin-tazobactam was deemed microbiologically inappropriate. In the setting of Japanese hospitals in our study, nearly 80% of patients who received either piperacillin-tazobactam or a carbapenem as initial therapy were able to have their antibiotics switched to narrow-spectrum antibiotics for definitive therapy; POC Gram stains allowed for clinicians to make these decisions more quickly. Thus, more evidence is accumulating in favor of using Gram stains at POC to reduce the use of unnecessarily broad-spectrum antibiotic therapy for complicated pyelonephritis.

Our study has 5 limitations. First, the study employed a retrospective, single-center design. However, our institution has established a routine protocol for collecting comprehensive data, including blood culture collection, urine Gram stains, and immediate drainage followed by submission of RPU. Second, there is a problem regarding the external validity in PCGS, and the data from our facility may not be generalizable across all healthcare settings. Although there are facilities with established feedback systems for PCGS in other research, there is no standardized educational program for PCGS in Japanese teaching hospitals [10]. However, it is reported that practical hands-on training, including workshops, can improve the accuracy of Gram stain interpretation [24]. Therefore, the educational progress of Gram stains, along with recognizing prevalent diagnostic errors and characteristics, could make PCGS a reliable diagnostic tool even for obstructive pyelonephritis.

Third, this study may not have obtained an adequate number of cases for comparison given that cases with mild hydronephrosis outnumber those with severe hydronephrosis.

Fourth, this study included a relatively small number of cases with negative urine culture results from either BU or RPU to accurately diagnose obstructive pyelonephritis. This may have potentially lowered the true-negative rates and affected calculations of specificity.

Fifth, we excluded 17 patients who did not receive POC Gram stains and 3 patients who died within 48 hours of admission, preventing the collection of appropriate data. Among these, there were sepsis and septic shock cases, and it is possible that some of these cases would have tested positive if Gram staining had been performed. By excluding these cases, there is a possibility that the results of the Gram staining were underestimated.

## CONCLUSIONS

Among cases with obstructive pyelonephritis, BU PCGS for GNRs demonstrated a sensitivity and specificity comparable to those obtained through laboratory testing in those with mild hydronephrosis, with a moderate to good level of agreement with urine culture results. Physicians should be aware of false-negative PCGS results, especially in cases with GPC in the urine or severe hydronephrosis, or if urine cultures are obtained from below the urinary tract obstruction where they may be falsely negative.

## Supplementary Material

ofae026_Supplementary_Data

## Supplementary Data

Supplementary materials are available at *Open Forum Infectious Diseases* online. Consisting of data provided by the authors to benefit the reader, the posted materials are not copyedited and are the sole responsibility of the authors, so questions or comments should be addressed to the corresponding author.

## Notes

***Acknowledgments*.** We express our gratitude to all the interns, ED doctors, and internal medicine residents who performed Gram staining in the ED. We would also like to thank the staff of the bacteriology laboratory who conducted Gram staining, identification, and susceptibility tests.
