# Supplementary material for: Diagnostic Accuracy of Point-of-Care Gram Stains in Obstructive Pyelonephritis due to Ureteral Stones
Source: Open Forum Infect Dis. 2024 Feb 2;11(3):ofae026. doi: 10.1093/ofid/ofae026 (PMC10913829; doi:10.1093/ofid/ofae026)
Supplement: ofae026_Supplementary_Data [file ofae026_supplementary_data.zip › Supplement 3.docx]

| **Table S3. Diagnostic property and concordance rates of Gram staining for BU classified by degree of urine bacterial counts.** | | | | | | | | |
| --- | --- | --- | --- | --- | --- | --- | --- | --- |
| **GNR positive group in BU culture** | | | | | | |  |  |
|  | Sensitivity %, [95%CI] | Specificity %,  [95%CI] | PPV %,  [95%CI] | NPV %,  [95%CI] | Positive LR,  [95%CI] | Negative LR,  [95%CI] | Kappa  coefficient | Agreement % |
| POC |  |  |  |  |  |  |  |  |
| ≤10^3^ CFU/mL | 59.1 [36.4–79.3] | 88.5 [69.8–97.6] | 81.3 [54.4–96] | 71.9 [53.3–86.3] | 5.12 [1.67–15.7] | 0.46 [0.28–0.78] | 0.486 | 75 |
| ≥10^4^ CFU/mL | 92.1 [84.5–96.8] | 76.5  [50.1–93.2] | 95.3 [88.5–98.7] | 65 [40.8–84.6] | 3.93 [1.66–9.25] | 0.1  [0.05–0.22] | 0.64 | 89.6 |
| Laboratory test |  |  |  |  |  |  |  |  |
| ≤10^3^ CFU/mL | 63 [42.4–80.6] | 100 [87.7–100] | 100  [80.5–100] | 73.7 [56.9–86.6] | - | 0.37 [0.23–0.61] | 0.634 | 81.8 |
| ≥10^4^ CFU/mL | 98 [92.9–99.8] | 90.9  [70.8–98.9] | 98  [92.9–99.8] | 90.9 [70.8–98.9] | 10.8 [2.87–40.4] | 0.02 [0.01–0.09] | 0.889 | 96.7 |
|  |  |  |  |  |  |  |  |  |
| **GPC positive group in BU culture** | | | | | | |  |  |
|  | Sensitivity %, [95%CI] | Specificity %,  [95%CI] | PPV %,  [95%CI] | NPV %,  [95%CI] | Positive LR,  [95%CI] | Negative LR,  [95%CI] | Kappa coefficient | Agreement % |
| POC |  |  |  |  |  |  |  |  |
| ≤10^3^ CFU/mL | 16.7  [0.42–64.1] | 68.8 [53.7–81.3] | 6.25 [0.16–30.2] | 86.8  [71.9–95.6] | 0.5  [0.08–3.35] | 1.21  [0.81–1.82] | 0.214 | 83.3 |
| ≥10^4^ CFU/mL | 56.3 [29.9–80.2] | 84.6 [76.2–90.9] | 36 [18–57.5] | 92.6 [85.4–97] | 3.66 [1.96–6.83] | 0.52 [0.3–0.9] | 0.33 | 80.8 |
| Laboratory test |  |  |  |  |  |  |  |  |
| ≤10^3^ CFU/mL | 66.7 [22.3–95.7] | 93.9 [83.1–98.7] | 57.1 [18.4–90.1] | 95.8 [85.7–99.5] | 10.9 [3.17–37.4] | 0.36 [0.11–1.1] | 0.564 | 90.9 |
| ≥10^4^ CFU/mL | 100 [79.4–100] | 92.4 [85.5–96.7] | 66.7  [44.7–84.4] | 100 [96.3–100] | 13.1 [6.74–25.5] | - | 0.762 | 93.4 |
| BU=bladder urine, RPU=renal pelvis urine, GNR=gram negative rod, GPC=gram negative cocci, CI=confidence interval, PPV=positive predictive, NPV=negative predictive value, LR=likelihood ratio,  AUC=area under the curve, POC=point–of–care. | | | | | | |  |  |
