# Supplementary material for: Diagnostic Accuracy of Point-of-Care Gram Stains in Obstructive Pyelonephritis due to Ureteral Stones
Source: Open Forum Infect Dis. 2024 Feb 2;11(3):ofae026. doi: 10.1093/ofid/ofae026 (PMC10913829; doi:10.1093/ofid/ofae026)
Supplement: ofae026_Supplementary_Data [file ofae026_supplementary_data.zip › Supplement 1.docx]

**Table S1. Gram Staining and Urine Culture Results of Gram-Negative and Gram-Positive Rods in the Classification of Hydronephrosis Severity: A 2x2 Table**

| **Gram negative rod** |  |  |  |  |  |  |  |  |
| --- | --- | --- | --- | --- | --- | --- | --- | --- |
| **Mild hydronephrosis** | |  |  |  | **Severe hydronephrosis** | |  |  |
|  | **BU culture** | |  |  |  | **BU culture** | |  |
|  | **Positive** | **Negative** | **Total** |  |  | **Positive** | **Negative** | **Total** |
| POC Gram stain |  |  |  |  | POC Gram stain |  |  |  |
| Positive | 103 | 6 | 115 |  | Positive | 15 | 2 | 17 |
| Negative | 12 | 28 | 40 |  | Negative | 6 | 8 | 14 |
| Total | 115 | 34 | 149 |  | Total | 21 | 10 | 31 |
|  |  |  |  |  |  |  |  |  |
|  | **BU culture** | |  |  |  | **BU culture** | |  |
|  | **Positive** | **Negative** | **Total** |  |  | **Positive** | **Negative** | **Total** |
| Laboratory Gram stain |  |  |  |  | Laboratory Gram stain |  |  |  |
| Positive | 123 | 2 | 125 |  | Positive | 20 | 1 | 21 |
| Negative | 10 | 38 | 48 |  | Negative | 6 | 10 | 16 |
| Total | 133 | 40 | 173 |  | Total | 26 | 1 | 37 |
|  |  |  |  |  |  |  |  |  |
|  | **RPU culture** | |  |  |  | **RPU culture** | |  |
|  | **Positive** | **Negative** | **Total** |  |  | **Positive** | **Negative** | **Total** |
| POC Gram stain |  |  |  |  | POC Gram stain |  |  |  |
| Positive | 29 | 2 | 31 |  | Positive | 4 | 0 | 4 |
| Negative | 3 | 7 | 10 |  | Negative | 3 | 2 | 5 |
| Total | 32 | 9 | 41 |  | Total | 7 | 2 | 9 |
|  |  |  |  |  |  |  |  |  |
|  | **RPU urine culture** | |  |  |  | **RPU urine culture** | |  |
|  | **Positive** | **Negative** | **Total** |  |  | **Positive** | **Negative** | **Total** |
| Laboratory Gram stain |  |  |  |  | Laboratory Gram stain |  |  |  |
| Positive | 53 | 2 | 55 |  | Positive | 12 | 0 | 12 |
| Negative | 5 | 15 | 20 |  | Negative | 4 | 7 | 11 |
| Total | 58 | 17 | 75 |  | Total | 16 | 7 | 23 |

| **Gram positive coccus** | |  |  |  |  |  |  |  |
| --- | --- | --- | --- | --- | --- | --- | --- | --- |
| **Mild hydronephrosis** | |  |  |  | **Severe hydronephrosis** | |  |  |
|  | **BU culture** | |  |  |  | **BU culture** | |  |
|  | **Positive** | **Negative** | **Total** |  |  | **Positive** | **Negative** | **Total** |
| POC Gram stain |  |  |  |  | POC Gram stain |  |  |  |
| Positive | 16 | 23 | 39 |  | Positive | 7 | 1 | 8 |
| Negative | 9 | 101 | 110 |  | Negative | 3 | 20 | 23 |
| Total | 25 | 124 | 149 |  | Total | 10 | 21 | 31 |
|  |  |  |  |  |  |  |  |  |
|  | **BU culture** | |  |  |  | **BU culture** | |  |
|  | **Positive** | **Negative** | **Total** |  |  | **Positive** | **Negative** | **Total** |
| Laboratory Gram stain |  |  |  |  | Laboratory Gram stain |  |  |  |
| Positive | 31 | 15 | 46 |  | Positive | 8 | 1 | 9 |
| Negative | 3 | 124 | 127 |  | Negative | 2 | 26 | 28 |
| Total | 34 | 139 | 173 |  | Total | 7 | 27 | 37 |
|  |  |  |  |  |  |  |  |  |
|  | **RPU culture** | |  |  |  | **RPU culture** | |  |
|  | **Positive** | **Negative** | **Total** |  |  | **Positive** | **Negative** | **Total** |
| POC Gram stain |  |  |  |  | POC Gram stain |  |  |  |
| Positive | 5 | 5 | 10 |  | Positive | 0 | 2 | 2 |
| Negative | 2 | 29 | 31 |  | Negative | 1 | 6 | 7 |
| Total | 7 | 34 | 41 |  | Total | 1 | 8 | 9 |
|  |  |  |  |  |  |  |  |  |
|  | **RPU culture** | |  |  |  | **RPU culture** | |  |
|  | **Positive** | **Negative** | **Total** |  |  | **Positive** | **Negative** | **Total** |
| Laboratory Gram stain |  |  |  |  | Laboratory Gram stain |  |  |  |
| Positive | 8 | 3 | 11 |  | Positive | 2 | 2 | 4 |
| Negative | 2 | 62 | 64 |  | Negative | 1 | 18 | 19 |
| Total | 10 | 65 | 75 |  | Total | 3 | 20 | 23 |
