# Supplementary material for: Diagnostic Accuracy of Point-of-Care Gram Stains in Obstructive Pyelonephritis due to Ureteral Stones
Source: Open Forum Infect Dis. 2024 Feb 2;11(3):ofae026. doi: 10.1093/ofid/ofae026 (PMC10913829; doi:10.1093/ofid/ofae026)
Supplement: ofae026_Supplementary_Data [file ofae026_supplementary_data.zip › Supplement 2.docx]

| **Table S2. Diagnostic property and concordance rates of Gram staining for BU classified by degree of hydronephrosis.** | | | | | | | | |
| --- | --- | --- | --- | --- | --- | --- | --- | --- |
|  |  |  |  |  |  |  |  |  |
| **GNR positive group in BU culture** | | | | | | |  |  |
|  | Sensitivity %, [95%CI] | Specificity %,  [95%CI] | PPV %,  [95%CI] | NPV %,  [95%CI] | Positive LR,  [95%CI] | Negative LR,  [95%CI] | Kappa  coefficient | Agreement % |
| **Mild hydronephrosis** | |  |  |  |  |  |  |  |
| POC | 89.6  [82.5–94.5] | 82.4 [65.5–93.2] | 94.5 [88.4–98] | 70 [53.5–83.4] | 5.08 [2.45–10.5] | 0.13 [0.07–0.22] | 0.677 | 87.9 |
| Laboratory | 92.5 [86.6–96.3] | 95 [83.1–99.4] | 98.4  [94.3–99.8] | 79.2 [65–89.5] | 18.5 [4.79–71.5] | 0.08  [0.04–0.14] | 0.818 | 93.1 |
| **Severe hydronephrosis** | |  |  |  |  |  |  |  |
| POC | 71.4 [47.8–88.7] | 80 [44.4–97.5] | 88.2 [63.6–98.5] | 57.1 [28.9–82.3] | 3.57 [1–12.7] | 0.36 [0.17–0.75] | 0.466 | 74.2 |
| Laboratory | 76.9 [56.4–91] | 90.9 [58.7–99.8] | 95.2  [76.2–99.9] | 62.5 [35.4–84.8] | 8.46  [1.29–55.5] | 0.25  [0.12–0.3] | 0.6 | 81.1 |
|  | |  |  |  |  |  |  |  |
| **GPC positive group in BU culture** | | | | | | |  |  |
|  | Sensitivity %, [95%CI] | Specificity %,  [95%CI] | PPV %,  [95%CI] | NPV %,  [95%CI] | Positive LR,  [95%CI] | Negative LR,  [95%CI] | Kappa coefficient | Agreement |
| **Mild hydronephrosis** | |  |  |  |  |  |  |  |
| POC | 64 [42.5–82] | 81.5  [73.5–87.9] | 41 [25.6–57.9] | 91.8  [85–96.2] | 3.45  [2.15–5.53] | 0.44  [0.26–0.75] | 0.372 | 78.5 |
| Laboratory | 91.2  [76.3–98.1] | 89.2 [82.8–93.8] | 67.4  [52–80.5] | 97.6  [93.3–99.5] | 8.45 [5.18–13.8] | 0.1  [0.03–0.29] | 0.709 | 89.6 |
| **Severe hydronephrosis** | |  |  |  |  |  |  |  |
| POC | 71.4 [29–96.3] | 85.3 [68.9–95] | 50  [18.7–81.3] | 93.5  [78.6–99.2] | 4.86  [1.91–12.4] | 0.34  [0.1–1.1] | 0.485 | 82.9 |
| Laboratory | 80  [44.4–97.5] | 96.3  [81–99.9] | 88.9  [51.8–99.7] | 92.9  [76.5–99.1] | 21.6  [3.08–152] | 0.21 [0.06–0.72] | 0.788 | 91.9 |
| MSU=midstream urine, GNR=gram negative rod, GPC=gram negative cocci, CI=confidence interval, PPV=positive predictive, NPV=negative predictive value, LR=likelihood ratio,  AUC=area under the curve, POCT=point–of–care–testing | | | | | | |  |  |
